# Silent Killer in the Nose: Two Cases of Nasal Alveolar Rhabdomyosarcoma in Adults

**DOI:** 10.7759/cureus.50430

**Published:** 2023-12-13

**Authors:** Ahmad Kamil Ahmad Fahmi, Syafiqah Kamel, Kanivannen Arasu, Chew Mianxin, Avatar Singh Mohan Singh

**Affiliations:** 1 Department of Otorhinolaryngology-Head & Neck Surgery, Taiping Hospital, Taiping, MYS; 2 Department of Pathology, Taiping Hospital, Taiping, MYS

**Keywords:** malignant soft tissue tumor, parameningeal tumor, small round blue cell, nasal mass, alveolar rhabdomyosarcoma

## Abstract

We report two cases of nasal alveolar rhabdomyosarcoma (ARMS) in adult patients from our center who presented with local mass effect and systemic involvement. Our first patient had spontaneous unilateral epistaxis. Her blood investigation showed severe thrombocytopenia, and the bone marrow biopsy result showed bone marrow infiltration by non-hematopoietic malignant cells. Nasal endoscopy showed a mass arising medial to the left middle turbinate. Our second patient presented with right eye proptosis, associated with blurring of vision. Nasal endoscopy showed a right whitish nasal mass arising lateral to the middle turbinate. Both patients were diagnosed by immunohistochemical analysis showing ARMS, a soft tissue malignancy uncommon in adults. RMS in adults has a worse prognosis. Hence, the management is challenging. Early diagnostic workup is essential for the commencement of early treatment for better oncological outcomes.

## Introduction

Rhabdomyosarcoma (RMS) is a soft tissue malignancy arising from premature mesenchymal cells. It is common in children and adolescents but exceedingly rare in adults [1]. Anatomically, it commonly arises in the extremities, followed by the trunk, genitourinary tract, head, and neck. RMS can be divided histopathologically into embryonal, pleomorphic, alveolar, and mixed types. It varies in the histological pattern, including poorly differentiated tumors, making timely diagnosis difficult without the help of immunohistochemical and molecular study. The most common subtype is embryonal, constituting about 60% of RMS cases [2]. The alveolar subtype consists of another 20% of RMS cases that are predominant in adolescents. Alveolar rhabdomyosarcoma (ARMS) is more aggressive and has a poorer prognosis [2].

## Case presentation

Case 1

**Figure 1:**
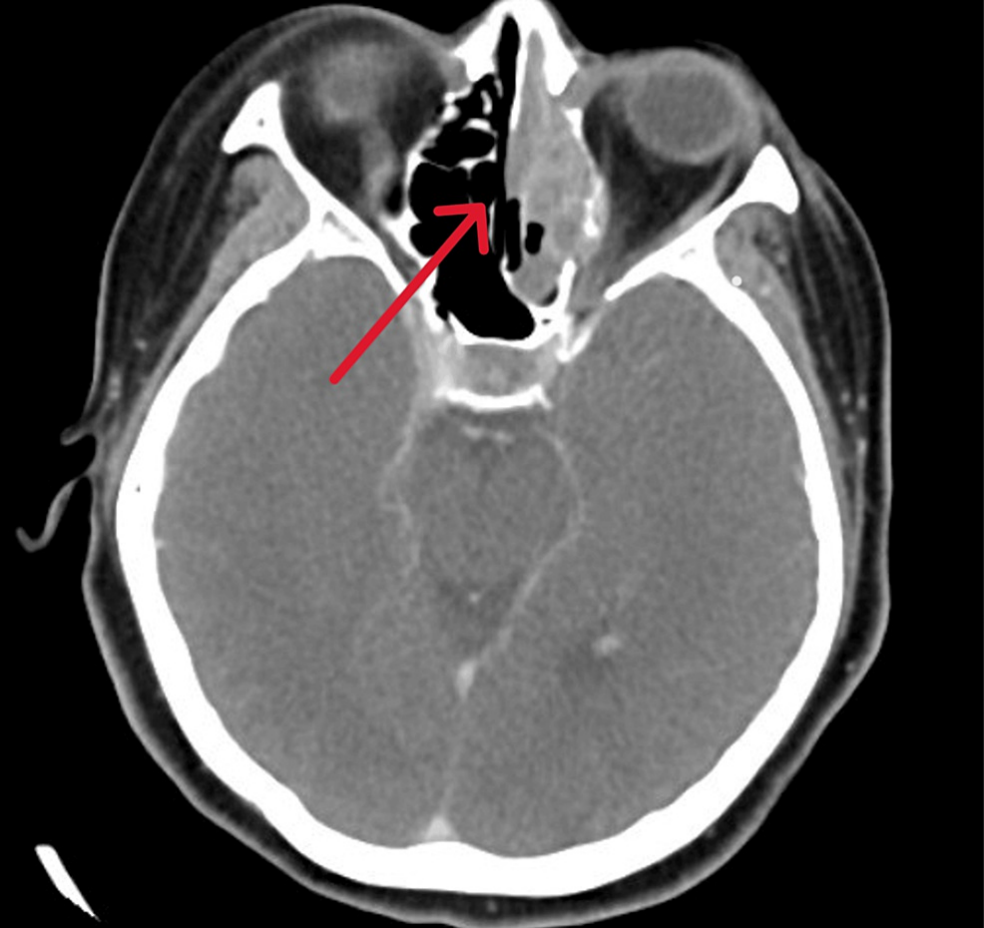
Case 1: CT axial view image shows a mass with its epicenter at the left anterior ethmoid (arrow)

A 55-year-old female with no known comorbid presented with a single episode of spontaneous unilateral epistaxis requiring anterior nasal packing to stop the bleeding. Her blood investigation showed severe thrombocytopenia, for which the patient received a platelet transfusion. However, the repeated thrombocytic count was persistently low. Subsequently, the patient underwent a bone marrow biopsy, revealing bone marrow infiltration by non-hematopoietic malignant cells. These malignant cells were immunoreactive to desmin and myogenin. On contrast-enhanced computed tomography (CT) imaging, a nasal mass was seen in the ethmoid and maxillary sinus with bony erosion and bone marrow infiltration (Figure 1), thus raising the possibility of primary malignancy in the nostril. Nasal endoscopy showed a reddish mass arising medial to the left middle turbinate. Nasal mass biopsy was taken for histopathological examination (HPE), which revealed alveolar rhabdomyosarcoma (ARMS). The patient subsequently rapidly deteriorated due to pancytopenia and systemic infection, leading to loss of life within days of diagnosis of her condition.

Case 2 

**Figure 2:**
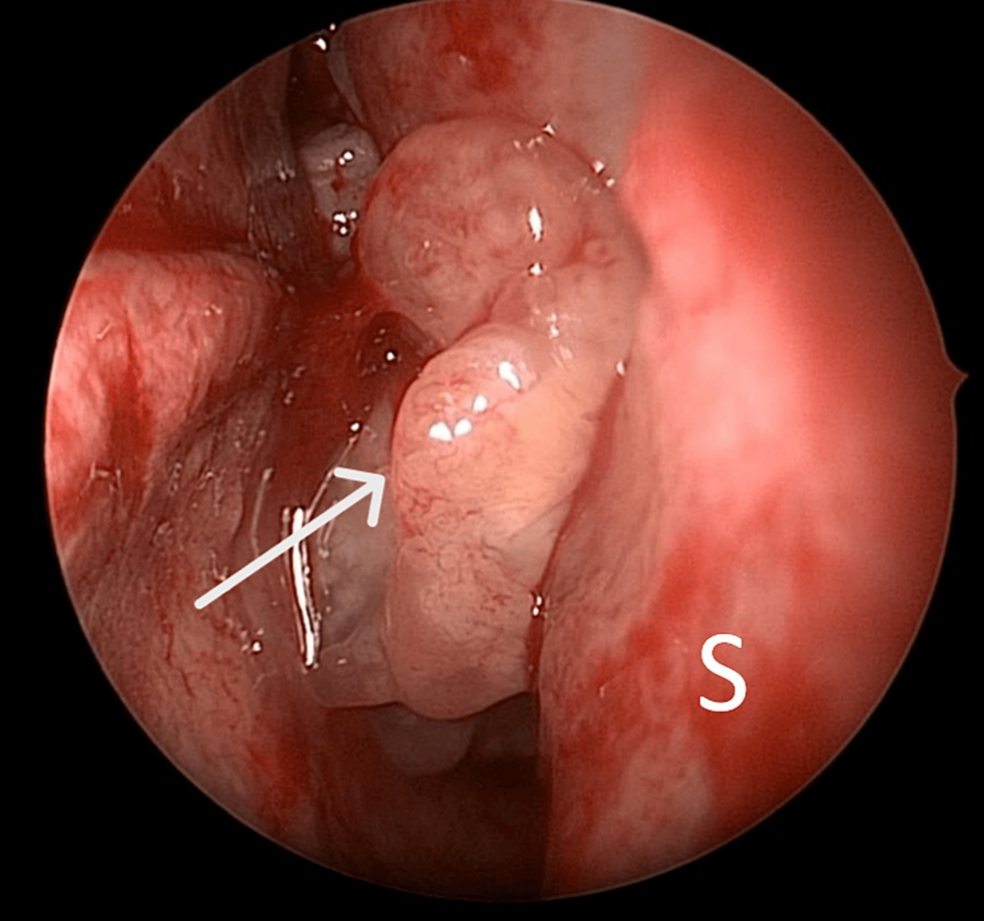
Case 2: The right nasal endoscope image shows the nasal mass (arrow) S = nasal septum

**Figure 3:**
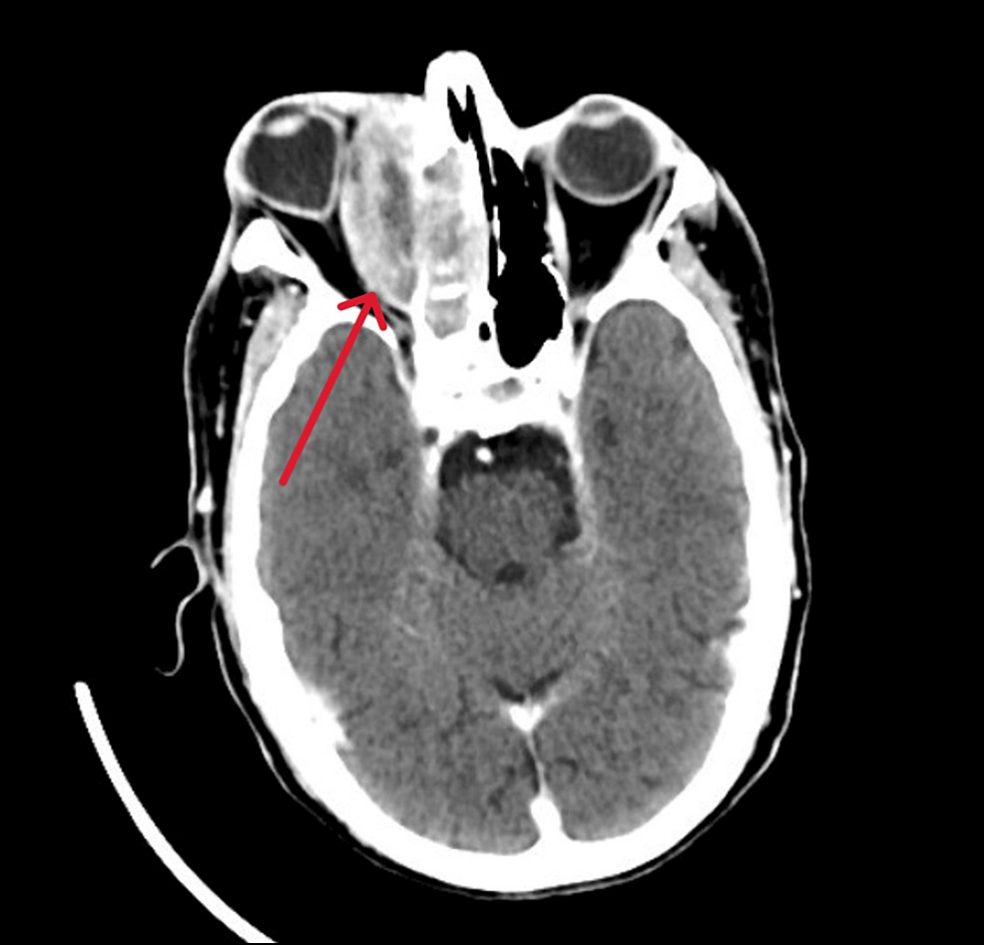
Case 2: Axial view CT shows the mass from the right nasal cavity extending into the orbital space (arrow). Noted also was a mass effect toward the orbit.

**Figure 4:**
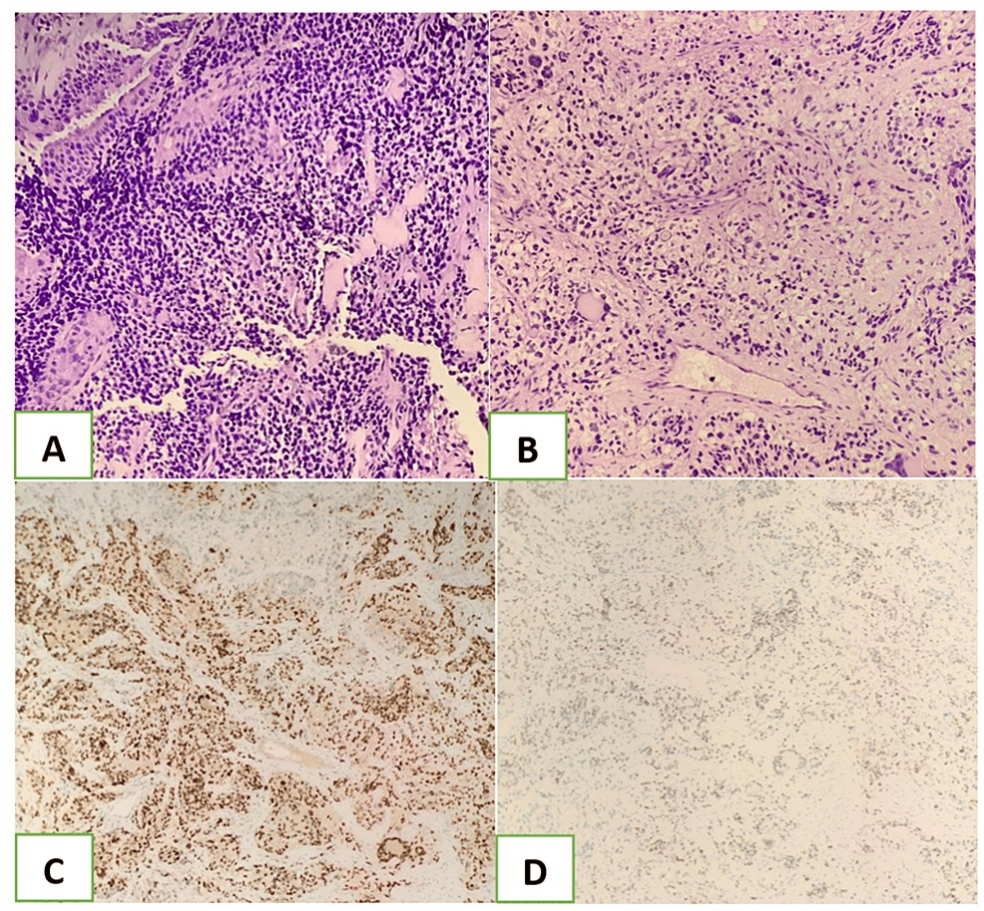
Case 2: HPE slides of alveolar rhabdomyosarcoma A) Primitive round blue cells dispersed in a solid pattern. B) Tumor cells with clear cytoplasm and occasional multinucleated giant tumor cells with a wreath-like lineup of nuclei. C) Diffuse and strong nuclear expression of myogenin. D) Diffuse and weak nuclear expression of MYOD1. HPE: histopathological examination

A 73-year-old male was referred to our team by the ophthalmology department given right eye protrusion associated with blurring of vision, giddiness, and vomiting. A complete physical examination revealed right eye proptosis with multiple ipsilateral neck nodes. Nasal endoscopy showed a whitish nasal mass arising lateral to the right middle turbinate and pushing the middle turbinate medially (Figure 2). Subsequently, contrast-enhanced CT imaging was arranged; it revealed a right nasal mass with orbital infiltration and proptosis (Figure 3). Histopathological examination (HPE) of the nasal mass showed alveolar rhabdomyosarcoma (Figure 4). The patient, however, refused surgical and oncological treatment due to advanced age and comorbidity. The patient succumbed to his disease.

## Discussion

RMS is a common soft tissue tumor in childhood and adolescence but rare in the adult population. It arises from primitive mesenchymal tissue with skeletal muscle lineage. It is the most common sarcoma in childhood, with around four to seven million annual incidents [3]. In adults, soft tissue sarcoma constitutes less than 1% of all adult malignancies, and RMS consists of 3% of all soft tissue sarcoma [4,5]. RMS can arise from any anatomic site of the body, with the highest prevalence in extremities, followed by the trunk, genitourinary tract, head, and neck [3].

Signs and symptoms depend on the primary anatomic site of the tumor. In the head and neck, it can arise from the orbit, parameningeal sites (middle ear, nasal cavity, paranasal sinuses, nasopharynx, and infratemporal fossa), and other sites (oral cavity, scalp, parotid gland, thyroid, pharynx, and neck region). Parameningeal tumors, such as nasal cavities, will produce nasal obstruction, epistaxis, and aural obstruction. The tumor will enlarge and produce a local mass effect such as proptosis and ophthalmoplegia with orbital involvement. Less than 25% of patients have metastasis at the time of diagnosis. The lung is the most frequent site, followed by bone, bone marrow, and lymph nodes [3].

The embryonic and alveolar subtypes are the two most prominent types of RMS. ARMS can be diagnosed through histopathologic examination in which tumor tissue shows undifferentiated small round blue cells lining up resembling pulmonary alveoli, hence the name “alveolar RMS.” Protein markers specific to muscle, such as alpha-actin, myogenin, myoglobin, desmin, Z-band protein, and Myo-D, are beneficial in identifying them histologically [3]. Other studies suggest histology remains an independent prognostic factor in which the alveolar subtype has poorer outcomes than the embryonic subtype tumor treated in an equivalent regime [6]. Electron microscopy may provide additional input in finding actin-myosin bundles or Z-band material to support a diagnosis of RMS. The molecular diagnostic method is becoming readily available in the evaluation of these tumors. The characteristic gene abnormality can be determined by reverse transcriptase polymerase chain reaction (RT-PCR), which provides a definitive ARMS diagnosis [7].

RMS is difficult to diagnose given its rarity, numerous primary anatomic sites, multiple histological types, and varied extent of disease at the time of diagnosis. The main components of evaluating suspected RMS include the extent of the primary disease and the presence of metastatic spread [3]. Besides, a thorough physical examination should include attention to the lymphatic system. Laboratory workup includes complete blood count, blood urea nitrogen, serum electrolytes, liver function test, calcium, magnesium, and phosphate. In some cases, bone marrow aspiration and biopsy of the iliac crest can provide vital information to achieve diagnosis. Radiologic assessment for possible metastatic spread should consider a chest CT and a technetium-99m diphosphonate bone scan. Any site that appears to be abnormal warrants to be investigated further.

As seen in these two current cases, ARMS behaves aggressively. It causes local mass effect and metastasis, which carries a poor prognosis. Treatment for RMS requires multimodal approaches, incorporating surgery, radiation therapy, and chemotherapy. Surgical resection, such as en bloc resection or wide excision, is considered complete when histologic margins are free if it is not mutilating or cosmetically damaging. In some cases where complete surgical resection is not feasible, neoadjuvant chemotherapy and local therapy are appropriate. Radiation therapy plays a vital role in treating RMS, such as of the head and neck, as tumors are not always resectable or completely excised. In general, radiation therapy guidelines are available in intergroup studies based on the staging and site of the tumor. In parameningeal tumors, the ability to define the radiation field and use 4,500-5,500 cGy have dramatically improved survival rates [3].

Chemotherapy is also important in treating RMS, especially if it is associated with micrometastatic disease. The development of adjuvant and neoadjuvant therapy increased the survival rate to approximately 60%. Some of the agents are vincristine (V), doxorubicin (Dox), actinomycin D (A), ifosfamide (I), cyclophosphamide (C), and etoposide (E). A combination of chemotherapy such as VAC, has been the gold standard in treating RMS. Patients with metastasis have an exceedingly poor prognosis despite being treated aggressively [5].

## Conclusions

Rhabdomyosarcoma (RMS) is a malignant soft tissue tumor consisting of a few subtypes, which is rare in adults and has a poor prognosis. Patients can present either with local or systemic problems, which may make it difficult to reach the diagnosis of RMS. Thorough physical examination and utilization of readily available diagnostic tools are essential in reaching an early diagnosis to ensure prompt treatment and better oncological outcomes.
